# Paradoxical Brain Herniation: An Unexpected Diagnosis

**DOI:** 10.7759/cureus.63723

**Published:** 2024-07-03

**Authors:** Inês M Amaral, Sérgio Sousa, Joana Martins, Heloísa Castro

**Affiliations:** 1 Intensive Care Unit, Unidade Local de Saúde do Santo António, Porto, PRT; 2 Neurosurgery, Unidade Local de Saúde do Santo António, Porto, PRT; 3 Critical Care, Unidade Local de Saúde Tâmega e Sousa, Penafiel, PRT

**Keywords:** neuroimaging and neurointervention, neurocritical care, adult intensive care unit, intensive care unit, neurology and critical care

## Abstract

Paradoxical herniation is a dreadful neurosurgical complication often underdiagnosed, which typically becomes evident over the course of weeks to months after the initial intervention. Here we present a unique case with manifestations in the post-operative period. A patient initially referred to neurosurgery for a meningioma underwent an uneventful surgical excision, followed by the transient placement of a lumbar drain for 48 hours. On the first post-operative day, the patient exhibited progressively altered neurological status, with corresponding imaging revealing a transfalcine herniation, necessitating emergent decompressive craniectomy. Despite the medical and surgical interventions, there were continuous signs of neurological and imaging worsening, with increase in herniation, which led to the diagnosis suspicion of a paradoxical brain herniation. Consequently, a rapid reversal of neurological deficits was observed after applying maneuvers to augment the intracranial pressure, followed by cranioplasty. This case illustrates the utmost importance of clinical suspicion for the uncommon complications of neurointerventions.

## Introduction

Every action is followed by a consequence, even when a medical treatment is applied to resolve another clinical condition. Given this, neurosurgical interventions carry potentially dreadful consequences, which should alert every clinician to the possibility of paradoxical herniation, especially when seemingly unexplainable events develop. This report describes the diagnostic process of a paradoxical herniation, which requires interventions opposite to the conventional treatment of brain herniations.

Emergent neurosurgical interventions, such as decompressive craniectomy (DC), are considered life-saving procedures for innumerable etiologies. DC has become the definitive surgical procedure to manage medically intractable rise in intracranial pressure, following proper neurocritical care [1-6]. This technique is designed to overcome the space constraints of the Monro-Kellie doctrine by removing part of the skull vault over a swollen brain, which increases the volume that the brain can occupy under the scalp and hence reduces intracranial pressure. However, it perturbs the cerebral blood and cerebrospinal fluid (CSF) flow dynamics. [1-2,4]. As a result, among potential complications, a mesodiencephalic herniation syndrome, termed paradoxical herniation of brain, is frequently unrecognized and underreported [7]. We present the case of a 48-year-old woman with a diagnosis of paradoxical brain herniation that developed in the first week post-neurosurgical intervention, earlier than previously reported in the literature (most commonly it develops one month following surgical intervention). In this case, there was a progressive neurological deterioration characterized by focal neurological deficits and depressed level of consciousness, with further medical interventions aimed at lowering intracranial pressure, as advocated for most herniation syndromes. In contrast to traditional treatments such as osmotherapy, CSF drainage, and hyperventilation, these measures will exacerbate paradoxical herniation by increasing the pressure gradient across the craniectomy defect [5-6,8]. To this extent, it is foremost to counter the external forces of atmospheric pressure by raising the intracranial pressure, in which specific maneuvers can be applied, such as the Trendelenburg position, hydration, clamping of CSF drainage, and discontinuation of hyperosmolar drainage. Indeed, only after the diagnosis, hypothesis of this syndrome was considered due to the improvement of neurological deficits when the patient was lying flat for imaging scans, and it was after the respective treatment applied that the first clinical improvement was observed. Significantly, the clinical presentation of this syndrome conveys a variable spectrum, ranging from nonspecific symptoms leading to diagnostic delay to acute neurological deterioration [6].

Therefore, this clinical case intends to illustrate the utmost importance of clinical suspicion regarding potential complications associated with neurointerventions, as well as the significance of integrating all the clinical data, correlating with pathophysiology, and acknowledging that our actions always have an impact.

## Case presentation

**Figure 1:**
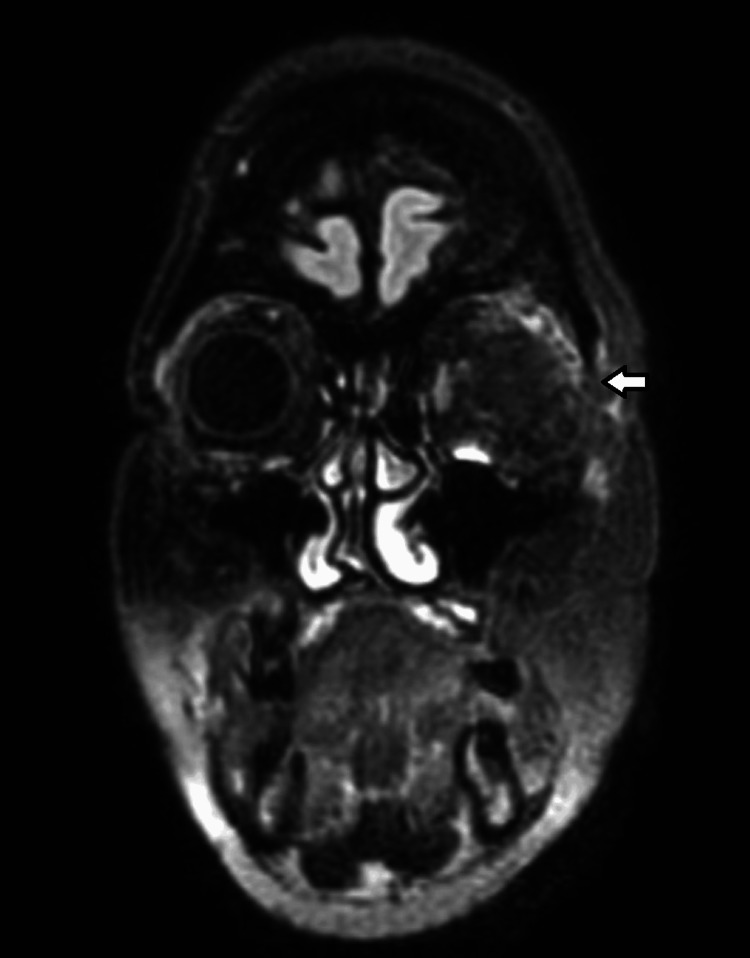
Pre-operative MRI FLAIR scan Meningioma of the left sphenoid wing, with projection into the temporal fossa and orbit, with diffuse meningeal extension to the temporal squama and left frontal bone. It reduces the left orbit, which compresses the muscles and optic nerve in the posterior part and causes proptosis.

**Figure 2:**
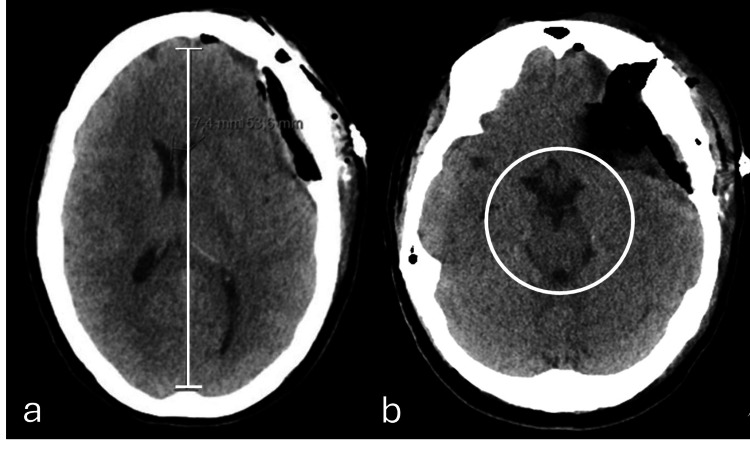
Axial plane of brain computed tomography obtained (a) post-meningioma resection (immediate post-operative phase) and (b) 24 hours post-resection without significant parenchymal changes (a) Signs of left fronto-temporal craniotomy associated with cranioplasty material (bone cement), covering a large part of the craniectomy area. It is also defined, underlying the craniotomy, as heterogeneous density material from the surgical duraplasty with small gaseous component (related to recent surgery) without significant hemorrhagic component. This material causes mass effect, with slight deformation of the adjacent parenchyma and ipsilateral ventricle, with 7 mm midline deviation of the midline structures to the right, but without significant subfalcine or uncal hernia. (b) The expansive effect exerted by these changes is unchanged, with persisting bihemispheric sulcal effacement and midline deviation of the structures to the right with attenuation of the suprasellar and perimesencephalic, which is associated with molding of the midbrain (white circle), without evident worsening compared to the prior (a) examination.

A 48-year-old female patient presented with a past medical history of strabismus and amblyopia in the left eye since childhood, resulting in reduced ipsilateral visual acuity. The patient also presented with a progressive proptosis of the left eye, evolving over approximately two years, accompanied by recent ocular pain, increased production of seromucous secretions, and a sensation of edema and pressure. Relative afferent pupillary defect on ophthalmology examination was found, and subsequent imaging was done by axial tomography of the orbits, with evidence of a lesion suggestive of intraosseous meningioma involving the superior temporal aspect of the orbit, small wing of the sphenoid and temporal scale, which was confirmed with magnetic resonance imaging, and diffuse meningeal capture extending to the temporal scale and left frontal bone (Figure 1). During follow-up, she was referred to neurosurgery with a diagnosis of left fronto-orbital meningioma requiring surgical excision. Subsequent elective surgery underwent uneventfully, with removal of the intracranial component of the left fronto-orbital meningioma extending almost to the base of the middle floor inferiorly and closed by replacing the non-invaded bone fragment with plates and screws. Immediately after the surgery, a lumbar drain was placed to aid dural plasty, which was removed after 48 hours. The immediate post-operative imaging did not show any unexpected abnormalities (Figure 2a).

On the first post-operative day, she developed dysphasia followed by transient altered neurological status with depressed level of consciousness (lethargic), which led to the suspicion of possible focal seizure activity by irritative phenomenon. After the introduction of anti-epileptic drugs (AEDs) and measures to reduce intracranial hypertension with osmotherapy, there was a slight clinical improvement in the patient’s awareness state but she remained dysphasic. Given the absence of imaging worsening (CT head with an expected left hemispheric surgical mass effect [Figure 2b] without any new acute lesions) and a compatible EEG examination with non-convulsive status, a dysphasic focal status epilepticus was assumed. However, on the second day, although being on triple AEDs on full dose (levetiracetam, lacosamide, phenytoin), the patient remained with fluctuating level of consciousness with progressive worsening to a stupor neurological state, which ultimately led for a therapeutic escalation with sedation. Following this, the patient was admitted to an intensive care unit.

Investigations and treatment

**Figure 3:**
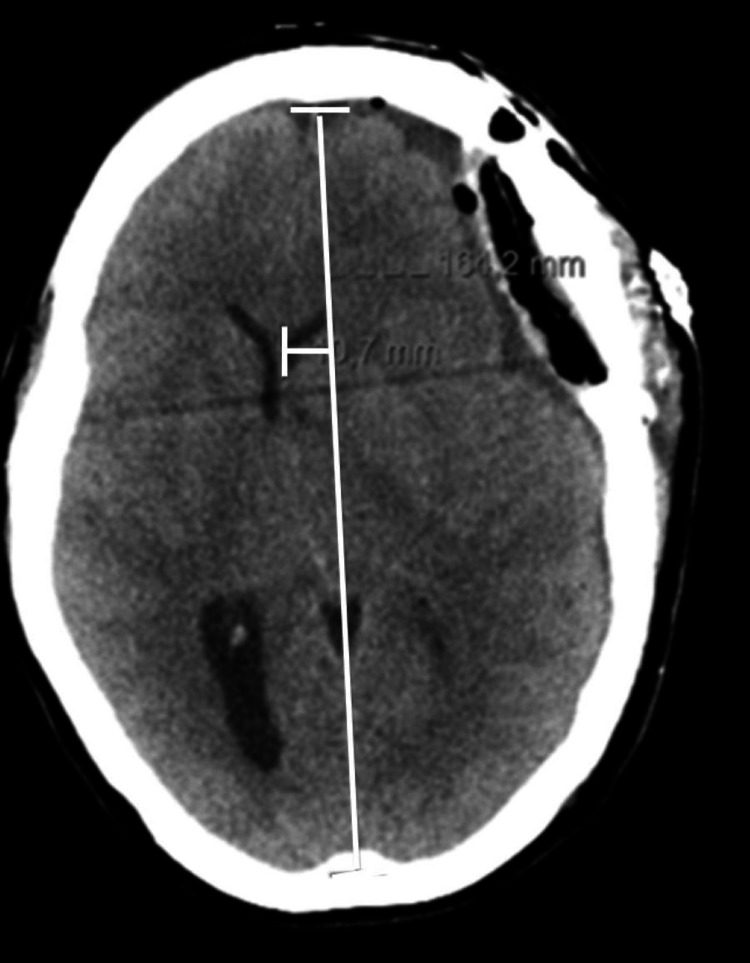
Axial plane of brain computed tomography at the third post-operative day and 24 hours under sedation A pericerebral hypodense collection remains, currently extending to the high fronto-parietal convexity. Consequently, there is a greater deformation of the brain parenchyma, resulting in a deviation of the midline to the right, currently approximately 11 mm, a degree greater than that shown in the previous examination (Figure 2).

**Figure 4:**
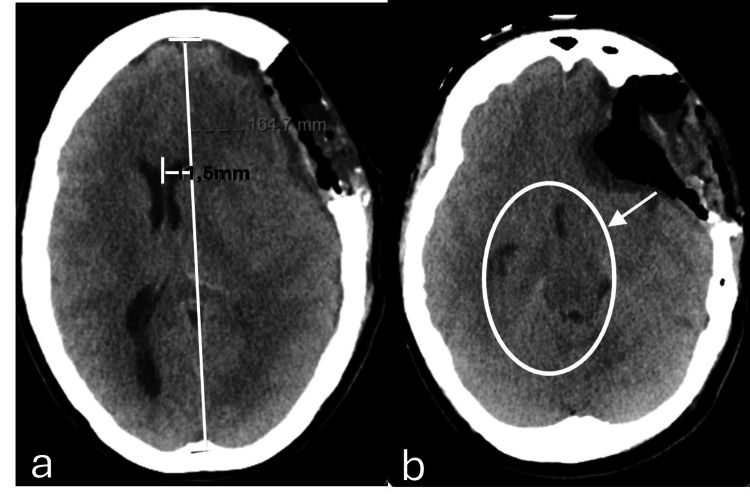
Axial plane of brain computed tomography after decompressive craniectomy (a) Mass effect remains on the left cerebral hemisphere and on the ventricular system, with signs of subfalcine hernia and clear deviation of the midline structures to the right, approximately 11.5 mm (the previous examination was approximately 10.7 mm). (b) An outline of an uncal hernia (white arrow), with molding of the midbrain and decreased permeability of the base cisterns (white circle).

At the time of admission in intensive care, the patient had already undergone extensive investigations, including an emergent angio-CT head imaging, with no remarkable acute abnormalities, and an EEG examination, with epileptiform waves indicating a probable nonconvulsive status epilepticus. Furthermore, the lumbar drain was removed after 48 hours post-surgery. To determine the underlying diagnosis for this clinical picture of altered neurological status with focal deficit characterized by sudden dysphasia, further imaging was pursued: after 24 hours under sedation and on the third post-operative day, revealed diffuse edema, predominantly in the temporal lobe, with a marked mass effect with transfalcine herniation with a midline shift of more than 10 mm and outline of a hernia from the uncus (Figure 3). In this context, an emergent DC was performed, with removal of the craniotomy flap. Nonetheless, on the fourth day, the patient developed anisocoria (left pupil bigger than the right), with subsequent tomography imaging (Figure 4) showing an increase in the mass effect associated with a significant midline shift (a 11.5 mm left to right midline shift vs the previous 10.7 mm), resulting in an extended craniectomy.

**Figure 5:**
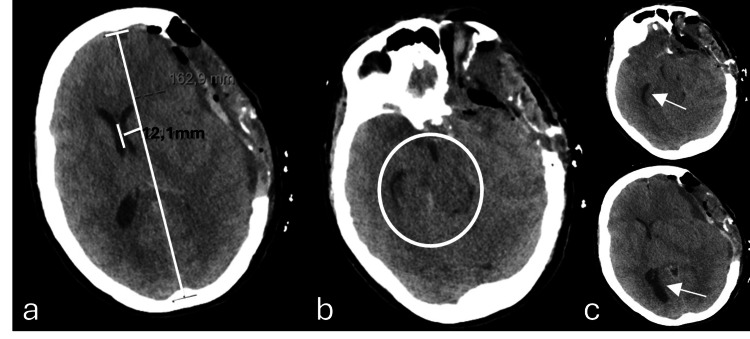
Axial plane of brain computed tomography taken 12 hours after post-craniectomy enlargement in the left temporoparietal region (third surgical intervention) due to neurological deterioration with fixed mydriasis There is evidence of bi-hemispheric convexity grooves effacement, base cisterns effacement, and 12 mm midline deviation to the right (a), as well as subfalcine herniation and inferior transtentorial herniation of the left uncus, with marked deformation of the midbrain (b). There is a slight increase in the amplitude of the temporal horn (superior arrow) and the occipital horn (inferior arrow) of the right lateral ventricle, reflecting an increase in internal CSF tension, due to disturbance of normal circulation (c).

Despite all the medical efforts to reduce the intracranial pressure and surgical interventions to mitigate the herniation effect observed, after 12 hours of the third operation, there were continuous signs of clinical deterioration, with new bilateral fixed mydriasis and new imaging demonstrating a progressive and worsening of the left to right midline shift (12 mm) and a successively more significant increase in paradoxical herniation (Figure 5). Paradoxically, intensive care physicians denoted a slight amelioration in pupillary response every time the patient underwent imaging evaluation, given the inevitable decrease of the angle of the bed to zero degrees and a reappearance of the pupillary abnormalities in returning to the unit and placed back to the recommended 30 degrees.

**Figure 6:**
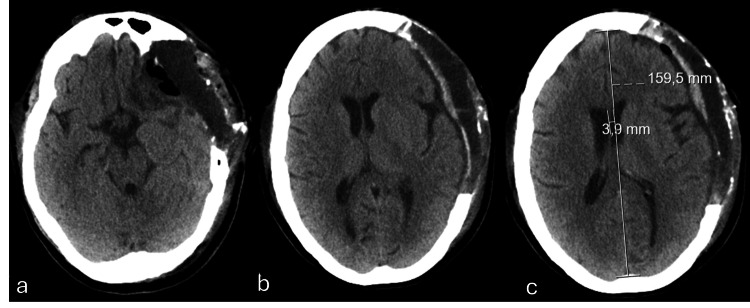
Axial plane of brain computed tomography obtained after adopting measures opposing intracranial hypotension There is a marked reduction in the existing anterior cerebral edema and consequent mass effect (a), with greater patency of the left hemispheric sulci and the lateral ventricle of the same side (b), persisting only with an incipient deviation of the structures from the midline, estimated at around 4mm to the right (c).

Therefore, in the presence of an unexplainable and continuous neurological deterioration refractory to all measures aimed at lowering intracranial pressure, integrating all the imaging findings (sustained and significant increased brain herniation away from the decompressed surgical site, causing subfalcine herniation, compressing the midbrain and effacement of the basal subarachnoid cisterns), and given the recent neurological intervention, including the presence of a lumbar drain in the immediate post-operative phase, paradoxical herniation was considered as the most likely diagnosis. Consequently, after assuming this diagnosis and promptly adopting measures that would oppose a decrease in the intracranial forces (in this case, supine position and optimizing hydration), a rapid clinical improvement with reversal of the neurological deficits (pupillary defects were resolved within minutes and consciousness level back to normal after sedation withdrawal) was observed, while maintaining the headrest at 0 degrees. Further investigation ensued, with new EEG displaying the absence of evidence of epileptiform activity and imaging studies unveiling substantial improvement of mass effect and parenchymal herniation reversal (Figure 6). Additional investigation was performed, following neurosurgery deliberation by the potential causative contribution of the lumbar drain for a CSF hypotension triggering the paradoxical herniation. Accordingly, a lumbar CT scan was performed with the exclusion of CSF fistula.

Outcome

**Figure 7:**
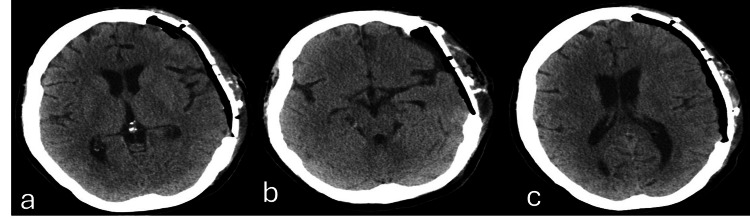
Axial plane of brain computed tomography obtained after cranioplasty Closure of the left fronto-temporo-parietal craniectomy area with cranioplasty plate – PEEK (a). Underlying the PEEK prosthesis there is a thin epidural collection with air component, without significant hemorrhagic component or a significant mass effect on the endocranial structures (b, c). PEEK, polyetheretherketone

**Figure 8:**
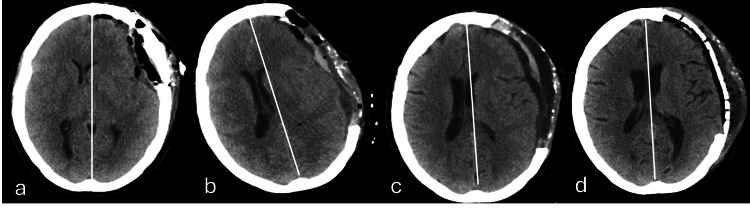
Chronological axial plane of brain computed tomography obtained throughout the clinical course (a) Post-meningioma resection. (b) Midline deviation of the structures to the right after decompressive craniectomy (herniation to the opposite site of the cranial defect). (c) Reduction of the herniation mass effect after adopting measures to increase the intracranial pressure. (d) Cranioplasty with plate (PEEK). The white line represents the midline. PEEK, polyetheretherketone

The patient remained in intensive care for 10 days in total and was transferred to the surgical ward. She remained hospitalized with progressive autonomy measures and physiotherapy. After 28 days from the first surgical intervention, the patient underwent cranioplasty with placement of a polyetheretherketone (PEEK) prosthesis, which is the gold standard treatment for this syndrome (Figures 7, 8). During the time she remained in the ward after this clinical scenario, she had a good clinical evolution, with CSF pocket in the retroauricular region stabilized, maintaining left proptosis and limited adduction of the left eye, but without evidence of motor lateralization. After 34 days of hospital stay, she was discharged to a continuous care facility to progress on her motor rehabilitation as a continuum of care initiated in the hospital.

## Discussion

Paradoxical brain herniation is an entity frequently underestimated and substantially underreported, with only a few case reports published in the literature. It is designated as “paradoxical” since it encompasses two paradoxical phenomena, there is a herniation of the decompressed side of the brain, contralateral to the cranial defect, and the treatment itself as it is exactly the opposite of the standard treatment approach applied to the vast majority of brain herniations [7].

This syndrome results from the barometric pressure difference between the atmospheric pressure and the intracranial pressure after removing a cranium segment, causing the brain to shift inward at the craniectomy site and affecting the CSF and cerebral blood flow dynamics. As these conditions represent low intracranial pressure states relative to extra-cranial pressures, it causes a brain herniation through the tentorial notch and potentially the foramen magnum. This paradoxical herniation is then defined as a midline deviation contralateral to the cranial defect, through the foramen magnum, causing compression and effacement of the midbrain [1-2,4-8]. While the main pathophysiologic mechanism is a direct transmission of atmospheric pressure to intracranial cavity, other features may also contribute to the clinical scenario, including decrease in cerebral blood flow resulting from microarterial circulation impairment, brain compression, and decrease in CSF pressure. This herniation pattern is more evident and susceptible for deterioration when there is a contribution for a ventricular collapse and alteration of the Monro-Kellie law, for instance after procedures involving CSF drainage, including a CSF leakage, ventricular shunt, or lumbar puncture in the postoperative period [4,7-9]. Indeed, in a patient with a major cranial defect, the CSF pressure in the lumbar region on a vertical position is greater than that in a patient with an intact cranial vault.

The clinical presentation conveys a variable spectrum ranging from nonspecific symptoms leading to diagnostic delay to acute neurological deterioration, including neurological focal deficits, signs of brainstem release, autonomic instability, pupillary changes, consciousness impairment, memory disturbances, language and cognitive deficits, weakness, confusion, seizures, and lethargy [1-2,4-9]. These symptoms can worsen with orthostatism, and its manifestations can occur earlier after the surgical event to years-after. Diagnosis is mainly clinically (can present with a concave skin flap at the craniectomy site) associated with neuroimaging revealing brain herniation away from the craniectomy with mass effect. Contrary to the traditional treatments for most herniation syndromes aimed at lowering intracranial pressure, these measures will exacerbate paradoxical herniation by increasing the pressure gradient across the craniectomy defect [2,4-9]. Recalling the main mechanism, the driving force is in fact an intracranial hypotension as opposed to intracranial hypertension. To this extent, it is foremost to counter the external forces of atmospheric pressure by raising the intracranial pressure, in which specific maneuvers can be applied, such as Trendelenburg’s position, avoidance of intracerebral hypotension (hydration and fluid therapy), clamping of CSF drainage, and discontinuation of hyperosmolar drainage. During the horizontal or Trendelenburg’s position, the CSF movement from the spinal compartment into the intracranial space leads to ventricular filling, filling of concave decompression sites, and hence clinical improvement. Notwithstanding, cranioplasty is the validated and definitive procedure for the treatment of paradoxical brain herniation with early and complete neurological recovery [10]. It has been shown that after the cranioplasty, the lumbar CSF pressure returned to its normal levels [7].

The following case enlightens the necessity to have an open mind to a new “invisible” force, namely atmospheric pressure, exerted on DC patients. A concave, introflexed aspect of the craniectomy flap and/or even a sudden neurological deterioration after a continuous period of improvement or a fairly rapid regression in the immediately post-craniectomy status are considered diagnostic features and red flags to be mindful of this syndrome. Notwithstanding, the main literature reports the timeline for this syndrome as a delayed post-craniectomy complication, typically encountered in the weeks to months after original craniectomy [1,6,9]. Over the years, investigations have been indicating that clinical manifestations can appear as early as three days to as late as seven years (with an average of five months) [1]. Rationally, emergent neurosurgical procedures are typically performed in patients with life-threatening brain insults, and once it is resolved, most patients will undergo a cranioplasty to close the defect, which generally occurs days to weeks after the original craniectomy [3]. This imposes a greater risk of developing a pressure gradient across the soft tissues of the cranium with respect to atmospheric pressure due to the absence of the skull’s rigid protective structure. All of this entails a susceptibility for inappropriate movements of the brain tissue with minimal alterations of the CSF, such as CSF drainage, if the atmospheric pressure exceeds the intracranial pressure [9,11]. Accordingly, paradoxical herniation is typically encountered in the weeks to months after original craniectomy. This case not only places emphasis on a rare disease entity among craniectomy patients but also brings about awareness for an early development of this syndrome, as in the post-operative phase of the emergent neurosurgical intervention, with all the management and treatment implications emphasized above. In fact, there are only few references of cases reporting the occurrence of this herniation after craniectomy before the usual time [11-12]. One of the speculating etiologies for the rapid development of this complication in our patient may lie in the fact that the patient had a CSF disturbance with the placement of a lumbar drain. However, the lumbar drain was removed before the craniectomy, and further investigations did not disclose perceivable signs of a CSF leakage, although these were not fully extendable. Nevertheless, there have been a few cases reports depicting that paradoxical herniation may occur in the absence of CSF drainage; for example, the remote possibility of CSF loss during multiple operations should be considered [11,13-15]. Additionally, it is paramount to acknowledge the importance of an early reference of patients with paradoxical herniation to ICU for neurological status monitoring, and the highly symptomatic patients with evidence of brainstem compression should be transferred to centers with neurosurgery consultation. A multidisciplinary approach is fundamental to delivering the best clinical practice possible, in particular by reflecting, conjecturing, and documenting all the alterations witnessed by different specialties and combining the wide expertise of each in inferring the final diagnostic hypothesis.

## Conclusions

Neurosurgical interventions, considering those done in an emergent context, are fraught with multiple and non-trivial complications, entailing a careful clinician’s anticipation to proceed with an early medical and surgical management. Hereby, this case report highlights the importance of having a low diagnostic threshold for the presence of unusual forms of brain herniation early in the course of the clinical events. Although paradoxical herniation has been documented as a delayed complication in the literature, typically succeeding DCs in the subsequent weeks to months, we have described a more expeditious onset of this phenomenon without an indubitably known inciting event.

To date, there is no randomized trial to estimate properly the risk of this complication following DC and the best timing for cranioplasty to preempt this specific syndrome.
